# Effect of Different Collection Times of Dermal Fibroblast Conditioned Medium (DFCM) on In Vitro Re-Epithelialisation Process

**DOI:** 10.3390/biomedicines10123203

**Published:** 2022-12-09

**Authors:** Nurul ‘Izzah Abdul Ghani, Rabiatul Adawiyah Razali, Shiplu Roy Chowdhury, Mh Busra Fauzi, Aminuddin Bin Saim, Binti Haji Idrus Ruszymah, Manira Maarof

**Affiliations:** 1 Centre for Tissue Engineering and Regenerative Medicine, Faculty of Medicine, Universiti Kebangsaan Malaysia, Kuala Lumpur 56000, Malaysia; 2 KPJ Ampang Puteri Specialist Hospital, Ampang 68000, Selangor, Malaysia; 3 Department of Physiology, Faculty of Medicine, Universiti Kebangsaan Malaysia, Kuala Lumpur 56000, Malaysia

**Keywords:** re-epithelialisation, conditioned medium, wound healing, keratinocyte, fibroblasts

## Abstract

A key event in wound healing is re-epithelialisation, which is mainly regulated via paracrine signalling of cytokines, chemokines, and growth factors secreted by fibroblasts. Fibroblast-secreted factors can be collected from the used culture medium, known as dermal fibroblast conditioned medium (DFCM). The goal of this study was to optimise the culture condition to acquire DFCM and evaluate its effect on keratinocyte attachment, proliferation, migration, and differentiation. Confluent fibroblasts were cultured with serum-free keratinocyte-specific (DFCM-KM) and fibroblast-specific (DFCM-FM) medium at different incubation times (Days 1, 2, and 3). DFCM collected after 3 days of incubation (DFCM-KM-3 and DFCM-FM-3) contained a higher protein concentration compared to other days. Supplementation of DFCM-KM-3 enhanced keratinocyte attachment, while DFCM-FM-3 significantly increased the keratinocyte wound-healing rate, with an increment of keratinocyte area and collective cell migration, which was distinctly different from DFCM-KM-3 or control medium. Further analysis confirmed that the presence of calcium at higher concentrations in DFCM-FM facilitated the changes. The confluent dermal fibroblasts after 3 days of incubation with serum-free culture medium produced higher proteins in DFCM, resulting in enhanced in vitro re-epithelialisation. These results suggest that the delivery of DFCM could be a potential treatment strategy for wound healing.

## 1. Introduction

The skin is the largest organ of the body and serves as a protective barrier that prevents environmental and pathogen attacks, as well as the loss of moisture and heat. Once this protective barrier is broken, the usual process of wound healing immediately sets in. Wound healing involves four distinct phases, i.e., haemostasis, inflammation, proliferation (including re-epithelialisation), and tissue remodelling [1]. These processes require interactions between cells in the epidermal (i.e., keratinocytes) and dermal (i.e., fibroblasts) layers, with an important role played by secretory factors and extracellular matrix (ECM) proteins [2].

Re-epithelialisation is one of the most important phases in wound healing, involving the re-establishment of the epidermal layer [3]. The re-epithelialisation process is strongly regulated by secreted proteins in the wound environment. These secreted factors, which include ECM, growth factors, cytokines, and chemokines, are a group of specialised multifunctional proteins that play a vital role in promoting keratinocyte attachment, proliferation, migration, and the stimulation of collagen production by fibroblasts [4,5]. It is well known that, during re-epithelialisation, fibroblasts secrete many of these factors, such as keratinocyte growth factor (KGF), granulocyte–macrophage colony-stimulating factor (GM-CSF), interleukin-6 (IL-6), and fibroblast growth factor-10 (FGF-10), which regulate keratinocyte properties via paracrine signalling [6,7,8,9].

Multiple studies have demonstrated the beneficial effect of supplementation with wound-healing factors such as platelet-derived growth factor (PDGF), FGF, and GM-CSF in the healing process [10,11]. However, due to the complex nature of the wound-healing process, which requires the interaction of myriad factors, researchers have explored the potential of supplementing cell-secreted factors to promote wounds and as supplementary therapies in wound treatment [12,13,14,15]. Cell-secreted factors are collected from the waste medium of cultured cells, commonly known as conditioned medium (CM). CM can be collected from various cell types (such as embryonic stem cells, mesenchymal stem cells, dermal fibroblasts, etc.) under different culture conditions (such as hypoxic or normoxic conditions) in the presence or absence of serum in the culture medium [16,17]. A wide range of wound-healing factors has been detected in the CM [18]. Supplementation with CM has been shown to enhance wound-healing potential, using both in vitro and in vivo models, by enhancing cellular proliferation, differentiation, migration, collagen production, and angiogenesis [12,19]. In recent decades, extensive studies have demonstrated the stimulatory effect of CM on the wound-healing properties of dermal fibroblasts [20,21]. However, the effect of CM collected from different time points on the keratinocyte wound-healing properties, i.e., re-epithelialisation has not been studied comprehensively.

In a previous study, DFCM was prepared by culturing human dermal fibroblasts in keratinocyte-specific and fibroblast-specific serum-free culture medium, and was shown to enhance the expansion of keratinocytes in vitro 2D and 3D models [13,22]. In the current study, we aimed to define the suitable culture conditions (collection time) to acquire DFCM, and the effect of DFCM on the in vitro re-epithelialisation process by assessing keratinocyte attachment, proliferation, migration, differentiation, and the wound-healing rate.

## 2. Materials and Methods

This study was approved by the Universiti Kebangsaan Malaysia Medical Research and Ethics Committee (UKM 1.5.3.5/244/FF-232-2013). In this study, skin samples were collected as redundant tissue. Informed consent was obtained from the patient or legal guardian before surgery.

### 2.1. Isolation of Cells and Primary Culture

Skin samples (*n* = 6) were collected as redundant tissue from consenting patients before surgery and processed according to the protocol described elsewhere [21]. The skin was cleaned and washed with Dulbecco’s phosphate-buffered saline (DPBS; Gibco, Waltham, MA, USA) before being cut into small pieces. Then, skin was digested using 0.6% collagenase type I (Worthington, NJ, USA) solution in a shaker incubator for 4–5 h at 37 °C and subsequently treated with trypsin/EDTA (TE; Worthington, NJ, USA) for 10 min. Digested tissues containing both fibroblasts and keratinocytes were centrifuged (Eppendorf, Hamburg, Germany) and the pellet was suspended in a coculture medium (1:1 mixture of F12: DMEM, FD; Gibco) with 10% foetal bovine serum (FBS; Gibco, Waltham, MA, USA) and Epilife with growth supplement (Gibco)). The cells were seeded into 6-well plates (Greiner Bio-One, Monroe, NC, USA) and grown at 37 °C in a 5% CO_2_ incubator (Eppendorf, Hamburg, Germany). The culture medium was replaced every two days until cells reached 80–90% confluence. Subsequently, cocultured cells were treated with TrypLE Select (TS; Gibco) for 5 min to detach fibroblasts. A suspension containing TS and fibroblasts was collected from the wells and centrifuged at 5000× *g* rpm for 5 min. The pellet was suspended in FD+10% FBS and seeded into 75 cm^2^ flasks (Greiner Bio-One). Keratinocytes remaining on the culture plate were washed with DPBS and supplemented with Epilife for further expansion.

### 2.2. Preparation of Dermal Fibroblast Condition Medium (DFCM)

To prepare DFCM, human dermal fibroblasts (Passage 3; P3) were cultured in FD+10% FBS medium with a seeding density of 5000 cells/cm^2^. After reaching 100% confluence, the waste medium was discarded, and cells were washed twice with DPBS to remove excess medium. Serum-free keratinocyte-specific medium, i.e., Epilife, supplemented with growth factors (denoted here as KM) and fibroblast-specific medium, i.e., FD pure (denoted here as FM), were then added to the fibroblast culture. Cells were incubated at 37 °C in a 5% CO_2_ incubator for 1, 2, or 3 days before the waste mediums were collected as DFCMs, denoted as DFCM-KM or DFCM-FM, respectively. In this study, to avoid donor-to-donor variation, DFCM was prepared by using fibroblasts from three donors, pooled, and it was used throughout the experiment.

### 2.3. Bicinchoninic Acid (BCA) Assay

A BCA assay kit (Sigma-Aldrich, Burlington, MA, USA) was used to quantify the total protein concentration in DFCM. Bovine serum albumin (BSA) was used as a protein standard and prepared in the range of 200–1000 µg/mL in a 96-well plate. The working reagent was prepared by mixing 50 parts of BCA solution with one part of 4% copper (II) sulphate pentahydrate solution. All samples and the standard were incubated with the working reagent for 30 min at 37 °C, and absorbance was measured at 562 nm using a spectrophotometer (Bio-Tek, Winooski, VT, USA), and the amount of protein in DFCM was calculated based on the standard curve.

### 2.4. Multiplex ELISA

Multiplex ELISA was performed to evaluate and quantify the presence of 19 different wound-healing mediators, including cytokines, chemokines, and growth factors (Table 1) in DFCM-KM-3 and DFCM-FM-3. The Procarta immunoassay kit (Affymetrix Inc., Santa Clara, CA, USA) was used according to the manufacturer’s instructions. In brief, DFCM (50 µL/well) was added to the well containing 19 different antibody-conjugated beads and incubated for 60 min. Subsequently, biotinylated detection antibodies were added and incubated for another 30 min. The wells were then supplemented with streptavidin/PE and incubated for 30 min. The signal was detected on a Luminex instrument. The detection limit was between 1 and 10 ρg/mL for different proteins.

### 2.5. Keratinocyte Attachment and Growth

Keratinocytes at P2 were trypsinised and seeded at a seeding density of 1 × 10^4^ cells/cm^2^ on the 12-well plates (Greiner Bio-One) to analyse cell attachment and proliferation. The culture conditions are described in Table 2. Three technical replicates for each condition were tested for each sample (*n* = 6). In the control culture, cells were supplemented with Epilife-containing growth supplement, i.e., KM only. For the test sample, DFCM was supplemented in a ratio of 1:3 to KM. To evaluate cell attachment and growth, keratinocytes were incubated at 37 °C in a 5% CO_2_ incubator mounted on a Nikon A1R-A1 microscope (Minato, Tokyo, Japan). Five random positions per well were selected, and images were captured every 20 min intervals up to 72 h. Cell numbers at 24 h were counted manually to determine the concentration of adherent cells using Equation (1):

(1) $$\mathrm{Concentration}\;\mathrm{of}\;\mathrm{Adherent}\;\mathrm{Cells}=\frac{\mathrm{Average}\;\mathrm{cell}\;\mathrm{count}}{{\mathrm{Area}\;\mathrm{of}\;\mathrm{the}\;\mathrm{image}\;\mathrm{in}\;\mathrm{cm}}^{2}}$$

The keratinocytes’ growth rate was evaluated by counting cell numbers at 24 and 72 h. The growth rate was evaluated using Equation (2).

(2) $${\mathrm{Growth}\;\mathrm{rate}\;(h}^{-1})=\frac{\mathrm{Ln}\;(\mathrm{Cell}\;\mathrm{concentration}\;\mathrm{at}\;72\;h/\mathrm{Cell}\;\mathrm{concentration}\;\mathrm{at}\;24\;h)}{\left(72\;h-24\;h\right)}$$

### 2.6. Single-Cell Migration

Single-cell migration was evaluated to determine the migration rate of keratinocytes under subconfluent conditions. As described in the previous section, time-lapse images were captured from five randomly selected positions per well. Cell migration was analysed using NIS Element AR 3.1 software (Nikon, Minato City, Tokyo, Japan). At least 150 cells from each condition were chosen randomly and tracked every 20 min for 1 h (36 h after seeding). The migration rate of the cells was evaluated every 20 min using Equation (3).

(3) $$\mathrm{Migration}\;\mathrm{rate}=\frac{{\sqrt{{\left(x_{2}{-x}_{1}\right)}^{2}+{\left(y_{2}-y_{1}\right)}^{2}}}^{}}{t_{2}-t_{1}}$$

where (x_1_, y_1_) and (x_2_, y_2_) indicate the centre of the cell evaluated by the software at time t_1_ and t_2_, respectively. The overall migration rate for a cell was evaluated by averaging migration rate for three 20 min segments.

### 2.7. Scratch Assay

The scratch assay was performed to determine the rate at which cells re-populated the scratch area. It was performed by scoring a confluent monolayer keratinocyte (P3) with a 10 µL pipette tip to create a wound area. After washing with DPBS, cells were supplemented with culture medium with or without DFCM. The images were captured every 20 min for 48 h using a time-lapse imaging system. Three technical replicates were performed for each condition for each sample (*n* = 6). The wound area was analysed using NIS Element AR 3.1 software, and the rate of healing under each condition was evaluated using the following equation:

(4) $$\mathrm{Rate}\;\mathrm{of}\;\mathrm{healing}=\frac{\mathrm{Initial}\;\mathrm{area}\;\left({\mu m}^{2\;}\right)-\mathrm{Final}\;\mathrm{area}\;\left({\mu m}^{2\;}\right)}{\mathrm{Observation}\;\mathrm{time}\;\left(h\right)}]$$

### 2.8. Dialysis

Dialysis of DFCM-FM-3 was done using a Mini Dialysis Kit (GE Healthcare, UK) to remove small molecules (<1 kD). The dialysis tube and cap were rinsed with distilled water. DFCM-FM-3 was added to the tube. The dialysis tube was inverted into a beaker containing DPBS without antibiotics overnight at 4 °C. The tube was spun in a magnetic stirrer briefly before collecting the dialysed DFCM-FM-3, denoted as DFCM-FM-3 (dialysis).

### 2.9. Concentration of Calcium

The concentration of calcium in DFCM-KM-3, DFCM-FM-3 and DFCM-FM-3 (dialysis) was evaluated using Cobas C 501 reagent (Roche Diagnostics, Washington, DC, USA). DFCM was transferred into the appropriate Cobas C 501 reagent cartridge, and then the cartridge was loaded for reading.

### 2.10. Immunocytochemical Staining

Cells were fixed with 4% paraformaldehyde and permeabilised with Triton X-100 before blocking of nonspecific binding sites with 10% goat serum. Then, the cells were incubated overnight with rabbit monoclonal [SP6] anti-Ki67 (Abcam, Cambridge, UK) or anti-Vinculin (Abcam, Cambridge, UK) or anti- Integrin-β1 (Abcam, Cambridge, UK) at 4 °C, followed by incubation with Alexa Fluor 594 goat anti-rabbit IgG (1:200; Molecular Probes, Eugene, OR, USA) for 2 h at 37 °C. Cells were counterstained with DAPI to visualise nuclei and phalloidin to visualise actin. Images were captured using a Nikon confocal laser scanning microscope.

### 2.11. Statistical Analysis

All quantitative data are presented as mean ± standard error of the mean (SEM) and were analysed using the Statistical Package for Social Sciences (SPSS, version 20.0). Statistical differences were evaluated using one-way analysis of variance (ANOVA) with Bonferroni post hoc analysis for three or more groups. Differences were considered significant at *p* ≤ 0.05.

## 3. Results

### 3.1. Morphology of Cells and Concentration of Proteins in DFCM

**Figure 1:**
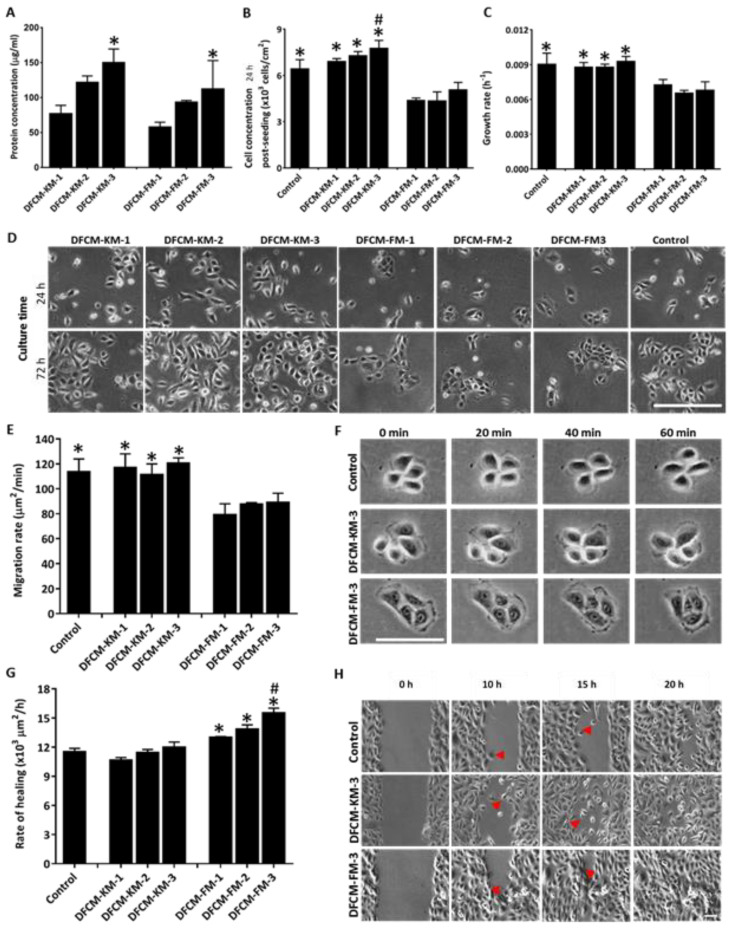
Preparation of DFCM and evaluation of its effects on keratinocytes re-epithelialisation. (**A**) Concentration of total protein in DFCM. Here, * indicates concentration of protein in DFCM-KM−3 and DFCM-FM−3 at 3 days incubation was significantly higher compared to that at 1-day incubation (*p* ≤ 0.05). (**B**) Effect of different DFCM on keratinocyte attachment evaluated 24 h after cell seeding. Here, * indicates significantly higher compared to DFCM-FM; # indicates significantly higher compared to control and DFCM-FM (*p* ≤ 0.05). (**C**) Keratinocyte growth rate under different culture conditions. Here, * indicates significantly higher compared to DFCM-FM; # indicates significantly higher compared to control and DFCM-FM (*p* ≤ 0.05). (**D**) Representative images of keratinocytes under culture conditions supplemented with either DFCM-KM−3 or DFCM-FM−3 compared with control for 24 h and 72 h of culture time. (**E**) Migration rate of keratinocytes under culture conditions supplemented with either DFCM-KM−3 or DFCM-FM−3 compared with control (*n* = 6). Here, * indicates significantly higher compared to DFCM-FM (*p* ≤ 0.05). (**F**) Representative images of keratinocytes for DFCM-KM−3, DFCM-FM−3, and control at 0, 20, 40, and 60 min to demonstrate single-cell migration. Arrow shows the movement of a cell. Scale bar: 100 µm. (**G**) Rate of healing of keratinocytes under culture conditions supplemented with either DFCM-KM or DFCM-FM compared with control (*n* = 6). Here, * indicates significantly different compared to control and DFCM-KM; # indicates significantly higher compared to other conditions (*p* ≤ 0.05). (**H**) Representative image of keratinocyte migration for DFCM-KM−3, DFCM-FM−3, and control during in vitro healing. Arrow demonstrates the individual or collective migration of keratinocytes during wound closure. Arrow indicates wound closure. Scale bar: 50 µm.

The fibroblast cells maintained their spindle shape (elongated) morphology from Day 1 to Day 3 in FM medium prior to collection of DFCM. However, the morphology of fibroblast starts to shrink at Day 3 whenever incubated with serum-free KM medium. The concentration of total protein in different DFCM (listed in Table 2) was evaluated using the BCA assay. It was found that the concentration of protein increased in DFCM-KM and DFCM-FM, along with an increase in the incubation time (Figure 1A). The protein concentration in DFCM-KM-−3 and DFCM-FM-3 was 150.28 ± 18.97 µg/mL and 112.16 ± 40.63 µg/mL, respectively, which was significantly higher than that of DFCM-KM−1 and DFCM-FM−1, respectively.

### 3.2. Effect of DFCM on In Vitro Re-Epithelialisation

DFCM-KM and DFCM-FM were collected after different incubation times (Table 2) and added to cultured keratinocytes to evaluate cell attachment, growth, and migration. Keratinocyte attachment was evaluated 24 h after seeding, and the growth rate was assessed between 24 to 72 h of culture. Keratinocyte migration was evaluated by live imaging under subconfluent (single-cell migration) and confluent conditions (wound healing). The results demonstrate that supplementation with DFCM-KM had no effect on keratinocyte properties except for attachment in DFCM-KM−3, which led to significantly greater attachment (77.60 × 10^2^ ± 5.15 × 10^2^ cells/cm^2^) compared to all conditions (Figure 1B,C). In contrast, supplementation with DFCM-FM resulted in a significant decrease in keratinocyte attachment, growth, and single-cell migration compared to DFCM-KM and control (Figure 1B–F). Surprisingly, supplementation with DFCM-FM significantly enhanced the keratinocyte wound-healing rate compared to the control and DFCM-KM conditions (Figure 1G,H). The highest healing rate was (155.38 ± 4.67) × 10^2^ µm^2^/h in DFCM-FM−3 and was significantly different compared to all other conditions. Moreover, a prominent difference was observed in the migration pattern, where keratinocytes in DFCM-FM supplemented conditions migrated collectively as a sheet, whereas under the control and DFCM-KM supplemented conditions, cells migrated individually during wound closure. Taking all these results together, subsequent experiments were performed using DFCM-KM−3 and DFCM-FM−3.

### 3.3. Identification of Wound-Healing Mediators in DFCM

**Figure 2:**
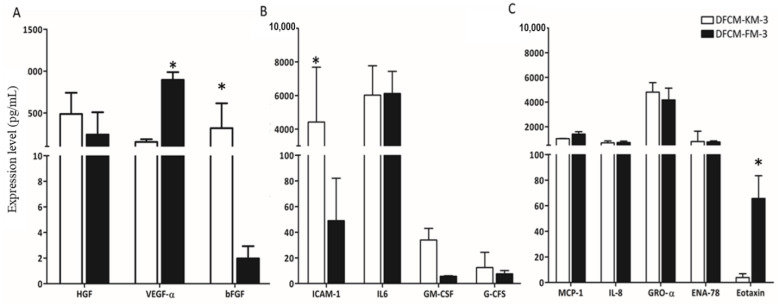
Wound-healing mediators on DFCM. (**A**) Growth factors, (**B**) cytokines, and (**C**) chemokines in DFCM quantified by multiplex ELISA. Here, * indicates significantly different compared to respective culture condition.

Multiplex ELISA was performed to investigate the presence of 19 different cytokines, chemokines, and growth factors in the DFCM-KM−3 and DFCM-FM-3. However, only 12 wound-healing mediators were detected (Figure 2), including four cytokines (IL-6, ICAM-1, GM-CSF, and G-CSF), five chemokines (MCP-1, IL-8, GRO-α, ENA-78, and Eotaxin), and three growth factors (HGF, VEGF-α, and bFGF). Among the detected factors, the concentration of ICAM-1 was significantly higher in DFCM-KM−3 compared to DFCM-FM-3, whereas the concentration of VEGF-α was significantly higher in DFCM-FM−3 compared to DFCM-KM-−3. No significant differences were observed for the other detected factors.

### 3.4. Expression and Distribution of Actin, Vinculin, and Integrin in Keratinocytes Treated with DFCM

**Figure 3:**
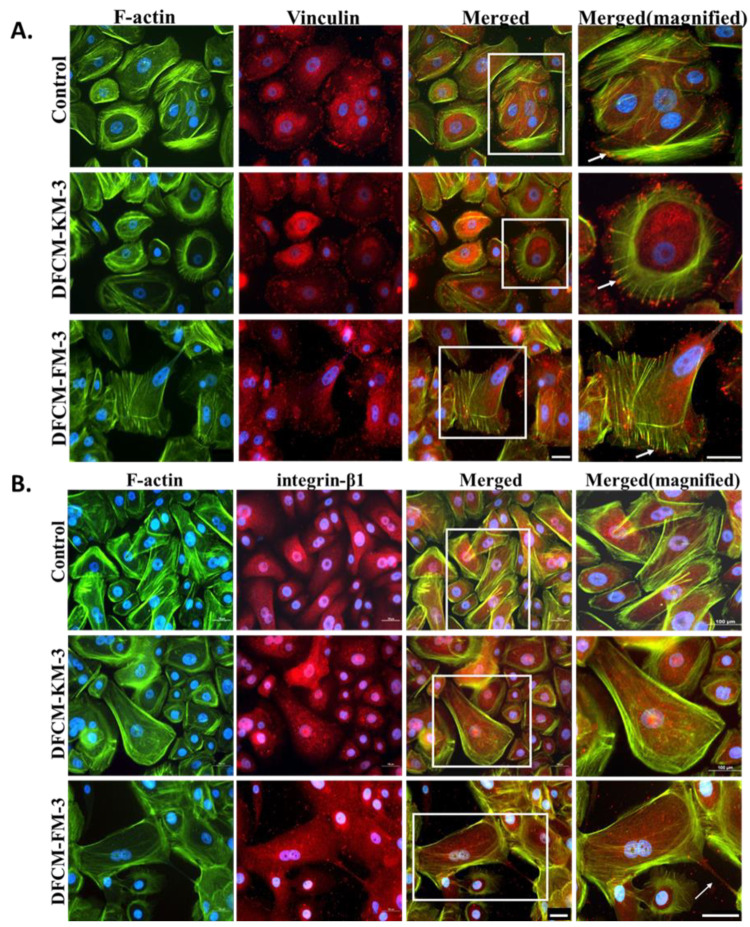
ICC staining of the keratinocyte with vinculin and integrin-β1 marker. (**A**) Representative images of keratinocytes stained with F-actin (green), DAPI (blue), and vinculin (red). Arrow shows expression of vinculin on the tip of stress fibres. Scale bar: was 100 µm. (**B**) Representative images of keratinocytes stained with F-actin (green), DAPI (blue), and integrin-β1 (red). Arrow shows filopodia with expression of integrin. Scale bar: 100 µm.

Actin and vinculin are cytoskeletal proteins associated with cell–cell and cell–matrix junctions, and morphological changes are strongly regulated by these cytoskeletal proteins. Immunocytochemical (ICC) staining showed that actin fibres were distributed at the periphery of the keratinocytes with or without DFCM; however, prominent stress fibres are visible in keratinocytes treated with DFCM-FM−3 (Figure 3A). In contrast, vinculin was diffusely distributed in the cytoplasm. It was also present in a cortical band outlining the cell borders, and present in focal contacts and adhesions. More stress fibres in keratinocytes were visible when supplemented with DFCM-FM−3 compared to DFCM-KM−3 and control. We also investigated the expression and distribution of integrin-β1 protein associated with cytoskeletal proteins that function in sensing cell adhesion to the ECM. Integrin-β1 was found in the cytoplasm of the keratinocyte, as well as motility structures such as filopodia and lamellipodia; however, no notable difference was observed among the culture conditions (Figure 3B). These results indicated that supplementation of DFCM-FM−3 demonstrated the formation of stress fibres with more focal adhesion.

### 3.5. Concentration of Calcium in DFCM

Calcium is a well-known regulator of keratinocyte properties. According to the manufacturer’s data sheet, FM contains more calcium compared to KM, which may have resulted in the flattening of keratinocytes along with the enlargement of cell structures when cells were supplemented with DFCM-FM. Thus, the calcium level in each DFCM was evaluated. It was found that DFCM-KM−3 contained only 0.06 mmol/L of calcium, while DFCM-FM-3 contained 1.08 mmol/L of calcium (Table 3). The dialysis of DFCM-FM−3 with a 1 kD cut-off tube efficiently removed calcium in DFCM-FM-3, and the calcium concentration was reduced to 0.01 mmol/L. Therefore, to investigate the role of calcium in keratinocyte proliferation potential, differentiation, morphology, and migration, cells were cultured with DFCM-KM−3, DFCM-FM−3, DFCM-KM−3 with the addition of 1.02 mml/L calcium chloride (denoted as DFCM-KM−3+CaCl₂), and DFCM-FM−3 after dialysis (denoted as DFCM-FM−3 (dialysis)) along with the control.

### 3.6. Effect of Calcium in DFCM on Keratinocyte Morphology

**Figure 4:**
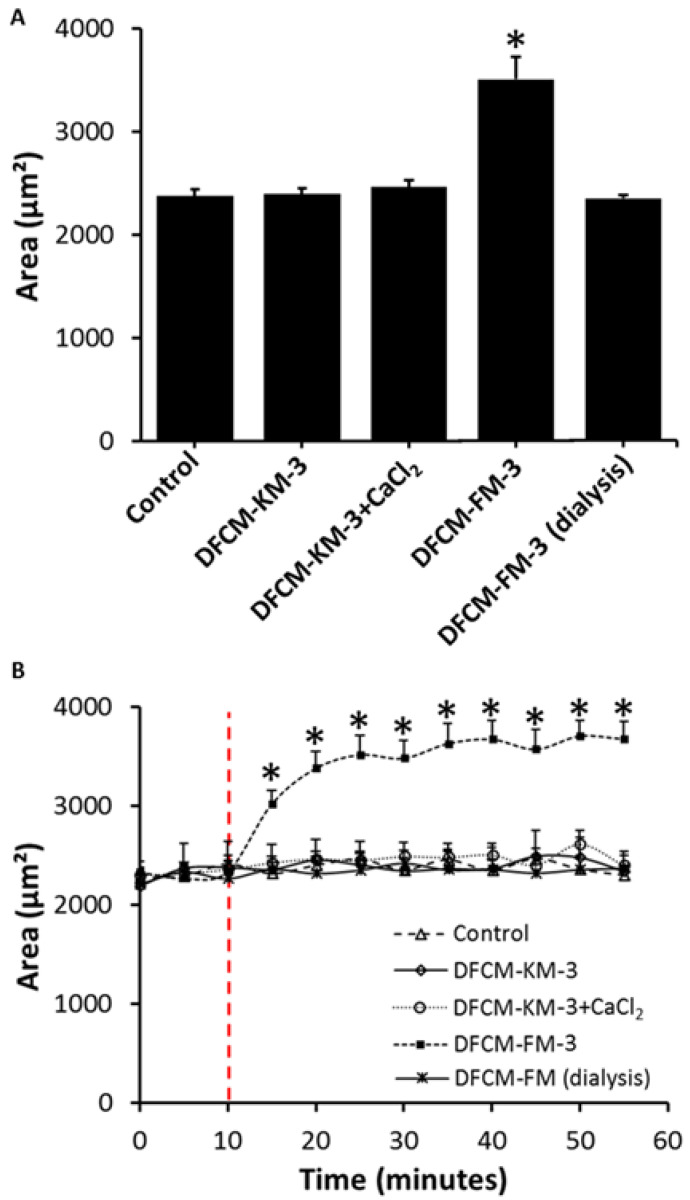
Effect of calcium in DFCM on keratinocyte area. (**A**) Keratinocyte area detected 24 h after supplementation with DFCM-KM−3, DFCM-FM−3, DFCM-KM−3-CaCl_2_, or DFCM-FM−3 (dialysis) in compared to control. (**B**) Quantitative evaluation of keratinocyte area before and after supplementation with DFCM. Here, * indicates significantly higher compared to other conditions (*p* ≤ 0.05).

Changes in keratinocyte area were evaluated 24 h after supplementation with DFCM. As shown in Figure 4A, the area of keratinocytes with DFCM-FM-3 supplementation (35.09 × 10^2^ ± 2.14 × 10^2^ µm^2^) was significantly higher compared to the control (23.78 × 10^2^ ± 0.65 × 10^2^ µm^2^) and DFCM-KM-3 (23.97 × 10^2^ ± 0.58 × 10^2^ µm^2^) cells. Interestingly, DFCM-FM-3 (dialysis), which contained a significantly lower level of calcium, led to a significantly smaller keratinocyte area (23.53 × 10^2^ ± 0.31 × 10^2^ µm^2^) than DFCM-FM-3. However, supplementation with DFCM-KM-3+CaCl_2_ resulted in no changes in keratinocyte area. In addition, to investigate the dynamic changes in keratinocyte area, time-lapse images of cells were captured at 5 min intervals, and the area of cells was measured. Keratinocytes were initially cultured in KM for 10 min, followed by supplementation with the different DFCM mentioned above, and observed for another 50 min. In the pre-supplemented condition, the keratinocyte area was 21.01 × 10^2^ ± 1.93 × 10^2^ µm^2^ (Figure 4B). However, the area of keratinocytes in the DFCM-FM−3 supplemented condition increased to 30.11 × 10^2^ ± 5.65 × 10^2^ µm^2^ within 5 min of supplementation. This increased further at 10 min to approximately 33.71 × 10^2^ ± 6.90 × 10^2^ µm^2^, with a slow increase thereafter. The area of keratinocytes supplemented with DFCM-FM−3 was significantly higher compared to the other conditions. There were no changes in the area of keratinocytes detected in the other conditions.

### 3.7. Effect of Calcium in DFCM on Keratinocyte Proliferation and Differentiation

**Figure 5:**
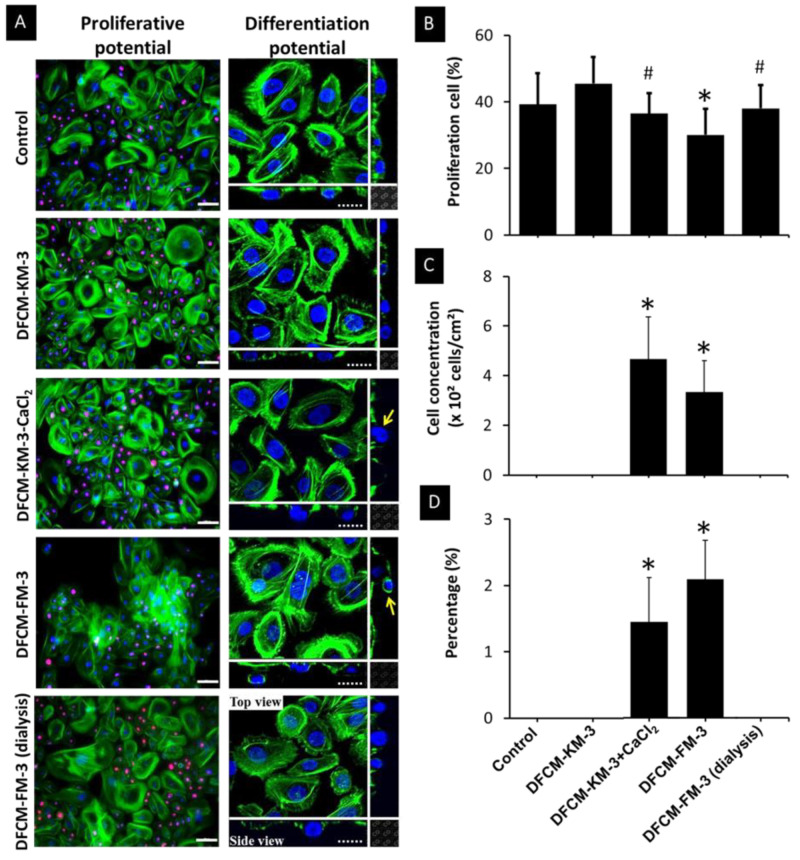
Effect of calcium in DFCM on keratinocyte proliferation and multilayer formation. (**A**) Representative images of actin (green), DAPI (blue) and Ki67 (red) expression by keratinocytes under different culture conditions. Arrow shows cells that formed multilayer. Scale bar: 100 µm. (**B**) Percentage of proliferating cells under different culture conditions. Here, * indicates significantly lower compared to control and other conditions; # indicates significantly lower compared to DFCM-KM−3 (*p* ≤ 0.05). (**C**) Cell concentration and (**D**) percentage of keratinocytes that formed a multilayer under different culture conditions. Here, * indicates significantly higher compared to other conditions (*p* ≤ 0.05).

The proliferation efficiency of keratinocytes was evaluated by staining with Ki67. Representative images of keratinocyte stained for Ki67 is shown in Figure 5A. Keratinocytes supplemented with DFCM-FM-3 showed significantly fewer proliferating cells (30 ± 8%) compared to the other conditions (Figure 5B). In the case of DFCM-FM−3 (dialysis), the proliferating cell number (38 ± 7%) was significantly increased over that in DFCM-FM−3. In contrast, DFCM-KM−3 + CaCl_2_ treatment led to a significant decrease in proliferating cell number to 36 ± 6% from 45 ± 8% (DFCM-KM−3) (Figure 5B). Additionally, it was noted that supplementation with DFCM-FM−3 and DFCM-KM−3 + CaCl_2_ led to the formation of keratinocyte multilayers, which was not detected in the other conditions (Figure 5A). A quantitative evaluation revealed that 4.67 × 10^2^ ± 1.69 × 10^2^ keratinocytes/cm^2^, which represents 2.09 ± 0.60% of total keratinocytes, formed a multilayer when supplemented with DFCM-FM−3. In the case of supplementation with DFCM-KM−3-CaCl_2_, the concentration of keratinocytes forming multilayers was 3.33 × 10^2^ ± 1.25 × 10^2^ cell/cm^2^, which represents 1.45 ± 0.67% of total keratinocytes (Figure 5C,D). Formation of a multilayer in keratinocytes indicates the initiation of a differentiation process. To confirm this differentiation efficiency, keratinocytes were stained with the terminal differentiation marker cytokeratin 10 (CK10). However, expression of CK10 was not observed under any conditions, even though DFCM-FM−3 and DFCM-KM−3+CaCl_2_ led to multilayer formation by keratinocytes.

### 3.8. Effect of Calcium in DFCM on Keratinocyte Migration

**Figure 6:**
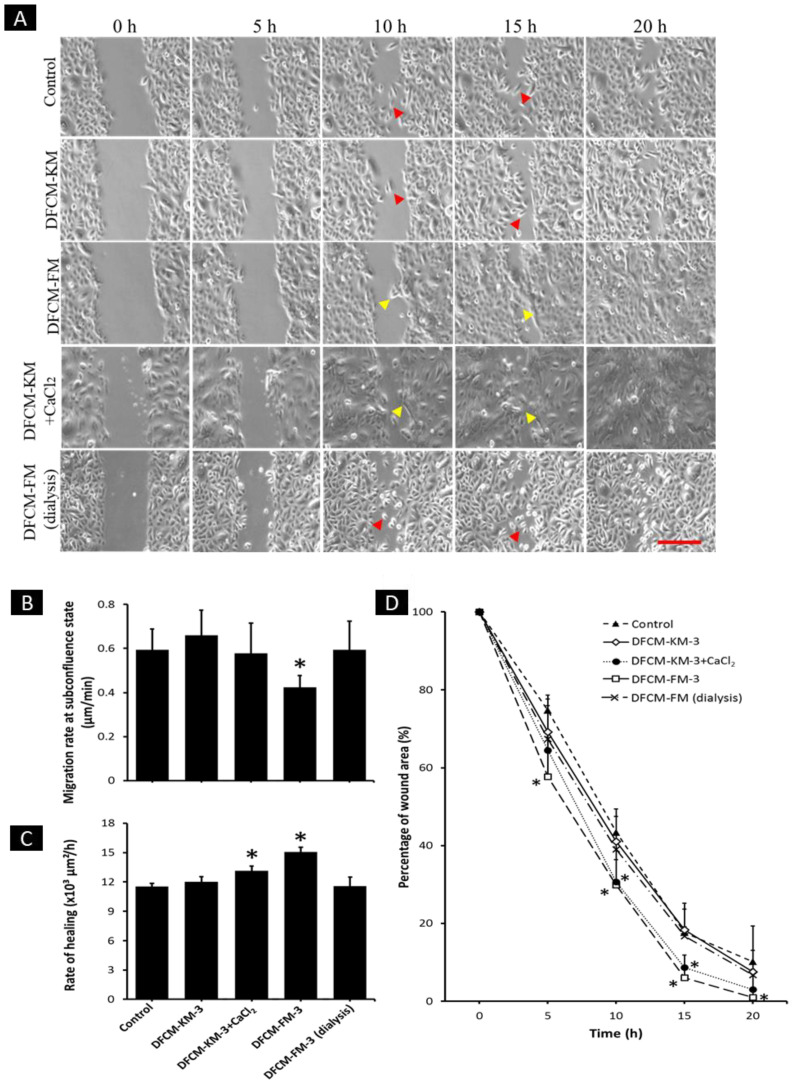
Effect of calcium in DFCM on keratinocyte migration under subconfluent and confluent conditions. (**A**) Representative image of keratinocyte migration during in vitro healing. Arrow demonstrates the individual (red) or collective (yellow) migration of keratinocyte during wound closure. Scale bar: 100 µm (red line) (**B**) Quantitative measurement of the keratinocyte migration rate under subconfluent conditions in culture supplemented with either DFCM-KM-3, DFCM-FM-3, DFCM-KM-3+CaCl₂ or DFCM-FM-3 (dialysis) compared with control. Here, * indicates significantly lower compared to other conditions (*p* ≤ 0.05). Effect of calcium in DFCM on keratinocyte migration during in vitro healing. (**C**) Rate of healing of keratinocytes under different culture conditions. (**D**) Progression of wound healing of keratinocytes in 5 h intervals for period of 20 h. Here, * indicates significantly different compared to control, supplementation of DFCM-KM-3 and DFCM-FM-3 (dialysis) (*p* ≤ 0.05).

As shown in Figure 6B, the migration rate of keratinocytes at subconfluence state was significantly lower after being supplemented with DFCM-FM−3 (0.43 ± 0.05 µm/min) compared to the other conditions. Interestingly, after supplementation with DFCM-FM-3 (dialysis), the migration rate increased significantly (0.59 ± 0.13 µm/min) compared to DFCM-FM−3; the value was comparable to control (0.60 ± 0.09 µm/min) and DFCM-KM-3 (0.66 ± 0.11 µm/min). Supplementation with DFCM-KM−3+CaCl_2_ slightly decreased the migration rate, but no significant difference was observed in comparison with the other conditions, except for DFCM-FM−3.

The rate of healing was evaluated using the scratch assay. As shown previously, supplementation with DFCM-FM−3 resulted in the collective migration of keratinocytes, while control and supplementation with DFCM-KM−3 led to individual cell migration during healing. The addition of CaCl_2_ in DFCM-KM−3 changed keratinocyte migration from individual to collective mode. In contrast, supplementation with DFCM-FM−3 (dialysis) resulted in a change in migration from collective to individual mode. In accordance with the previous results, the rate of healing in keratinocytes supplemented with DFCM-FM-3 was significantly higher compared to that in the control and DFCM-KM−3 supplemented cells (Figure 6A,C). Supplementation with DFCM-FM−3 (dialysis) resulted in a significant decrease in the healing rate (115.54 × 10^2^ ± 9.40 × 10^2^ µm^2^/h) compared to supplementation with DFCM-FM−3, while DFCM-KM−3+CaCl_2_ significantly increased the healing rate (131.53 × 10^2^ ± 4.80 × 10^2^ µm^2^/h) compared to supplementation with DFCM-KM−3.

For further understanding of the migration kinetics during healing, the migration rate of keratinocytes was evaluated at 5 h intervals up to 20 h (Figure 6D). The results show that supplementation with DFCM-FM−3 led to a significant reduction in wound area throughout the observation period compared to the other conditions, except for DFCM-KM−3+CaCl_2_. As expected, DFCM-KM-CaCl_2_ accelerated wound closure, with a significant decrease in the wound area at 10 h and 15 h compared to the other conditions, except for DFCM-FM−3.

### 3.9. Proliferative Potential of Keratinocytes during In Vitro Healing

**Figure 7:**
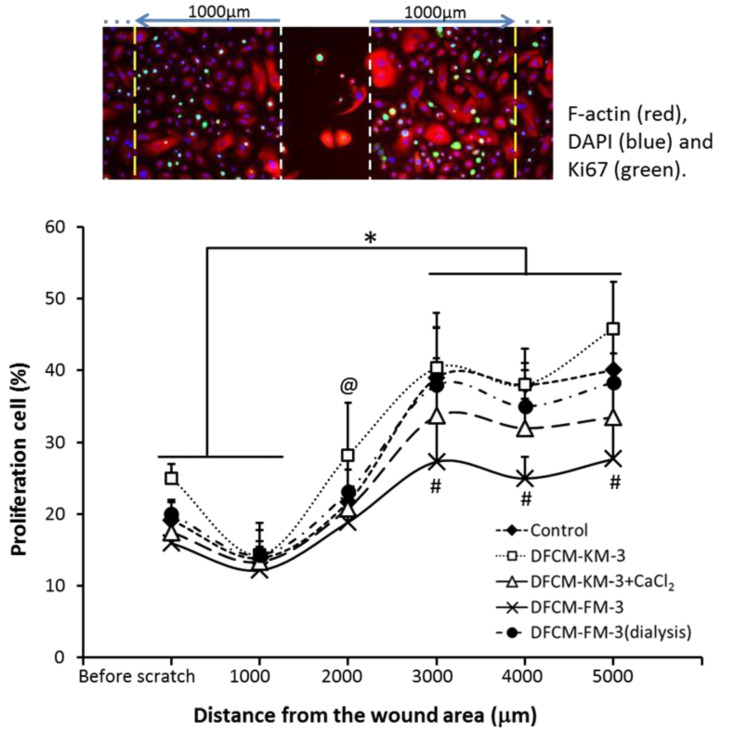
Percentage of proliferation cells from the edge of wound area up to 5000 µm region away from the wound under culture conditions supplemented with either DFCM-KM-3, DFCM-FM-3, DFCM-KM-3+CaCl₂ or DFCM-FM-3 (dialysis) compared with control. Here, * indicates significantly higher compared to the percentage of Ki67 before scratching for respective condition; # indicates significantly lower percentage of Ki67-positive cells in DFCM-FM-3 compared to control, DFCM-KM-3 and DFCM + KM-3 + CaCl_2_; and @ indicates significantly higher value in DFCM-KM-3 compared to other conditions; (*p* ≤ 0.05).

To investigate the contribution of keratinocyte proliferation during the healing process, the percentage of proliferating keratinocytes was analysed 12 h after wounding under different culture conditions. The percentage of Ki67-positive keratinocytes (proliferating cells) was determined for a region 5000 µm (step size was 1000 µm) from the edge of the wound (both sides). The results were compared to the percentage of proliferating cells before scratching (Figure 7). It was found that, after scratching, the percentage of proliferating cells was increased, especially in the region distal from the wound under all culture conditions. The percentage of proliferating cells close to the wound (within 1000 µm) was similar to that before scratching. However, the values increased exponentially within 3000 µm of the wound and stabilised thereafter. Although a similar trend was visible under all culture conditions, the percentage of proliferating cells decreased in the order: DFCM-KM−3 > control > DFCM-FM−3 (dialysis) > DFCM-KM−3+CaCl₂ > DFCM-FM−3.

## 4. Discussion

Previous studies have suggested that keratinocytes and fibroblasts function synergistically in vitro and in vivo. Fibroblasts have been shown to influence keratinocyte attachment, growth, proliferation, and migration during in vitro culture [23,24]. It has also been shown that fibroblast-secreted factors, as in dermal fibroblast conditioned medium (DFCM), facilitate keratinocyte attachment and expansion [13,25]. However, evidence for the effect of different DFCM collection times on keratinocytes is scarce. This is important to be explored to ensure the most optimum conditioned media with an abundance of wound-healing mediators is collected prior to use. In this study, we aimed to evaluate the potential of DFCM collected from different times on in vitro re-epithelialisation. It was found that fibroblast-secreted factors in serum-free KM and FM facilitate the in vitro re-epithelialisation process. DFCM-KM was shown to enhance keratinocyte attachment, whereas DFCM-FM increased keratinocyte migration during in vitro wound healing. Moreover, it was also found that calcium in DFCM-FM is one of the main factors enhancing keratinocyte migration during healing via collective migration.

In recent years, the potential of CM isolated from various cell types has been tested in wound healing, and has led to significant improvements [26]. CM has been prepared from cells using various culture conditions that differ in terms of passage number, number of cells, culture medium, and incubation time; these variables greatly affect the compositions of the CM and subsequent applications [26]. In this study, the focus was on optimising the culture conditions for fibroblasts, i.e., incubation time with two different types of serum-free medium to obtain DFCM. The quality of the DFCM was assessed based on the ability to promote in vitro re-epithelialisation of keratinocytes in terms of cell attachment, proliferation, migration, and wound-healing potential. It was shown that, by increasing the incubation time, the concentration of secreted proteins also increased, i.e., 3 days of incubation led to higher protein levels in both DFCM-KM and DFCM-FM compared to earlier time points. These results correlate with the in vitro re-epithelialisation properties of keratinocytes. Keratinocytes supplemented with DFCM collected after 3 days of incubation demonstrated significantly enhanced attachment and healing compared to media collected after shorter incubation times. These results suggest that a longer incubation time is suitable for producing secretory protein-enriched DFCM. However, an incubation time beyond 3 days was found to be inappropriate for fibroblast survival as serum-free medium was used to produce DFCM, especially the KM medium, which does not support the growth of fibroblast.

The secretion of wound-healing mediators plays a role in the response to skin loss [20,22]. The wound-healing process, which includes the homeostatic, inflammatory, proliferative, and remodelling phases, involved cross-talk communication between fibroblasts and keratinocytes [27,28]. Inflammatory cell signals that are released during the wound-healing process encourage fibroblast migration from the wound margins [29]. Basic fibroblast growth factor (bFGF/FGF-2) and vascular endothelial growth factor A (VEGF-A), which are paracrine factors secreted by fibroblasts, will signal nearby keratinocytes [27]. In addition, in response to paracrine signalling from keratinocytes and inflammatory cells, fibroblast will also synthesise collagen and differentiate into myofibroblastic phenotype [30]. Another example of a cross-talk event during wound healing is when keratinocytes secrete interleukin 1 (IL-1), which will promote keratinocyte migration and proliferation and mobilises neighbouring cells to promote healing. Subsequently, IL-1 will also stimulate fibroblasts’ synthesis of keratinocyte growth factor (KGF), which will aid in keratinocyte migration and proliferation [31]. This shows that the synergy relationship between fibroblast and keratinocytes is important in order to maintain tissue homeostasis and for the wound to heal completely.

In this study, secreted proteins such as cytokines, chemokines, and growth factors, which are directly involved in the re-epithelialisation processes, were identified in DFCM collected after 3 days of incubation since more protein concentration was found in both DFCMs at Day 3. Out of 19 wound-healing mediators, 12 were detected in both DFCMs, although the level of expression varied. The concentration of VEGF-α was significantly higher in DFCM-FM-3 compared to DFCM-KM-3, whereas the concentration of ICAM-1 was significantly higher in DFCM-KM-3 compared to DFCM-FM-3. These factors are mostly involved in angiogenesis, epithelialisation, and tissue remodelling processes [10,32,33,34,35,36,37,38], indicating that DFCM may facilitate the wound-healing process [39]. VEGF-α induces angiogenesis by stimulating endothelial cell mitogenesis [40]. Angiogenesis normally occurs during the proliferative phase of wound healing, and impaired angiogenesis may result in delayed wound healing. Additionally, skin wound healing is mediated by inflammatory cell infiltration, which is highly regulated by various adhesion molecules. The absence of ICAM-1 inhibits wound healing, keratinocyte migration from the edges of the wound toward the centre, and granulation tissue formation [41]. Previous studies have shown that mice lacking ICAM-1 demonstrate delayed skin wound healing [41].

The composition of the cell culture medium, which represents the in vitro microenvironment for cells in culture, plays a critical role in determining the composition of CM. Although some studies have used serum-containing medium, serum-free or serum-deprived media are more suitable as they stimulate the cells to secrete more proteins [42,43,44]. Moreover, this type of medium is more suitable for future therapeutic applications. In addition, the composition and concentration of organic and inorganic salts, amino acids, etc., in basal medium also determine the protein content in CM [45,46,47]. Our previous study on the proteomic analysis of both DFCM-KM and DFCM-FM has revealed that secretory proteins of DFCM are involved in cell adhesion, attachment, proliferation, and migration, which were demonstrated to have potential wound-healing effects by in vitro and in vivo studies [20]. In this study, it was found that using different basal media significantly affected the quality of the DFCM, reflected in keratinocyte properties. It was found that DFCM-KM enhanced keratinocyte attachment, while DFCM-FM enhanced keratinocyte migration during wound healing. It was also noted that DFCM-FM drastically changed keratinocyte morphology and led to collective cell migration, which was distinctly different from the DFCM-KM and control conditions. It was hypothesised that either the protein composition or the presence of small molecules resulted in these changes. However, the levels of predetermined wound-healing mediators were similar in DFCM-KM and DFCM-FM. Key differences cannot be concluded from this information, as CM contains numerous proteins, which requires an extensive proteomics study. Nevertheless, a careful comparison of the chemical composition of the basal media demonstrated a significant difference in the concentration of calcium in KM and FM, which was also confirmed by evaluating the concentration of calcium in DFCM-KM-3 and DFCM-FM-3.

Calcium is a well-known inducer of keratinocyte differentiation [48], and a calcium concentration >0.1 mM in culture triggers differentiation [49]. It was found that DFCM-FM-3 contained 1.08 mM calcium. As the keratinocyte culture medium was supplemented with 25% DFCM, the final concentration of calcium was approximately 0.27 mM, which is higher than the induction level for keratinocyte differentiation. A previous study demonstrated that calcium-dependent differentiation of keratinocytes resulted in rapid morphological changes along with the formation of cell–cell contacts [49]. In this study, supplementation with DFCM-FM led to similar phenomena. Thus, the effect of calcium in modulating the morphology, proliferation, differentiation, and migration of keratinocytes was tested.

Keratinocyte attachment, proliferation, and migration during re-epithelialisation are largely regulated by cytokines, chemokines, and growth factors from the provisional matrix and dermis. Calcium also plays a crucial role in cell attachment, proliferation, migration, and differentiation. In general, calcium facilitates cell attachment to a surface [50]; however, in this study, we found out that high calcium results in reduced cell attachment. The results of the present study show DFCM-FM led to a significant reduction in cell attachment. Moreover, DFCM-FM-3 and DFCM-KM-3-CaCl_2_ significantly reduced the proliferating cell number compared to control, DFCM-KM-3, and dialysed DFCM-FM-3. It was also noted that supplementation with DFCM-FM-3 and DFCM-KM-3-CaCl_2_ resulted in the formation of keratinocyte multilayers, indicating the initiation of differentiation, even though expression of CK10 was not observed.

Cell migration is mediated by the microenvironment, including the ECM, numerous secretory factors, and signalling molecules [51]. These factors regulate the dynamic organisation of the cytoskeleton (actin) to form focal adhesion points [52,53,54]. Supplementation with DFCM-KM facilitated single-cell migration of keratinocytes (under subconfluent conditions) compared to control and DFCM-FM. However, the addition of calcium to DFCM-KM-3 (DFCM-KM-3-CaCl_2_) resulted in a reduction in keratinocyte migration, while removing calcium from DFCM-FM-3 resulted in a significant increase in keratinocyte single-cell migration compared to their respective counterparts. This indicates that calcium is involved in the keratinocyte single-cell migration rate. This result is in accordance with a previous study by Fang et al. [55]. In contrast, the in vitro wound-healing rate was significantly higher when keratinocytes were supplemented with DFCM-FM-3 and DFCM-KM-3-CaCl_2_ compared to DFCM-KM-3, DFCM-FM-3 (dialysis) and control. This result demonstrates that, unlike single-cell migration, the presence of calcium facilitates keratinocyte migration during in vitro wound healing.

Previous studies have shown that extracellular calcium influx in keratinocytes is one of the earliest signals produced in response to wound generation and facilitates keratinocyte migration via cytoskeletal remodelling during healing [56,57]. However, a minimum level of calcium (<0.1 mM) is required to initiate the influx of extracellular calcium into keratinocytes, and cells are then dissociated from the wound edge towards the vacant area. In the current study, keratinocytes exposed to a low level of calcium (control, DFCM-FM-3 (dialysis), and DFCM-KM-3) also demonstrated the dissociation of cells from the wound edge. In contrast, keratinocytes exposed to a high level of calcium (approximately 0.27 mM in DFCM-FM-3 and DFCM-KM-3-CaCl_2_) underwent immediate flattening of cells with an enlarged cell area and demonstrated collective migration in a cell sheet. Similar patterns of keratinocyte migration during wound healing have been reported previously by Geer and Andreadis [58] when keratinocytes were cultured with serum. In this case, keratinocytes migrated via the formation of lamellipodia. During cell migration, lamellipodia projections formed by the polymerisation of actin undergo cycles of protrusion and retraction [59].

It was also noted that the changes in keratinocyte morphology and migratory properties induced by DFCM-FM-3 occur rapidly. Live cell imaging at 5 min intervals for 1 h demonstrated that the morphological changes occurred as early as 5 min after supplementation with DFCM-FM-3. However, these rapid changes in morphological properties were not observed when cells were supplemented with DFCM-KM-3-CaCl_2_, suggesting a synergistic effect with other small molecules. Together, these results provide evidence that calcium in DFCM plays a determining role in regulating keratinocyte migration during the in vitro re-epithelialisation process.

In wound healing, basal keratinocytes migrate from the wound edge to cover the injured area [60,61]. Once the wound area is covered, contact inhibition triggers their differentiation [60], indicating that migration and proliferation act in collaboration to facilitate wound healing. To understand the correlation between migration and proliferation in DFCM-modulated in vitro wound healing, the proliferative population of keratinocytes was determined. The evaluation was done not only at the edge of the wound but also away from the wound (up to 5000 µm). Interestingly, it was found that, under all conditions, the keratinocyte proliferative population was minimal at the edge of the wound but increased significantly away from the edge of the wound and reached a maximum around 3000 µm away from the edge and remained steady thereafter. Similar observations have been reported earlier, showing that keratinocytes at the rear of the wound margin multiply rapidly during wound healing [62,63]. However, in comparison with control and DFCM-KM-3, high calcium-containing DFCM, i.e., DFCM-FM-3 and DFCM-KM-3-CaCl_2_ showed a significantly lower number of proliferating cells. This result indicates that keratinocyte wound healing in the presence of DFCM-FM is different from that in DFCM-KM and control medium, where the contribution of keratinocyte proliferation is minimal.

## 5. Conclusions

In conclusion, confluent dermal fibroblasts after 3 days of incubation with serum-free culture medium produced DFCM, resulting in enhanced in vitro re-epithelialisation. However, it was revealed that supplementation with DFCM-KM enhanced keratinocyte attachment, but had no effect on proliferation and migration, while supplementation with DFCM-FM increased the keratinocyte wound-healing rate. The presence of calcium in DFCM-FM resulted in significant changes in keratinocyte morphological properties and migration patterns during wound healing. Together, these results suggest that delivery of DFCM could be a potential treatment strategy for wound healing. However, further investigation is required to standardise the production of CM and studies on the mode and effective dose delivery of CM for enhancing the re-epithelialisation process.

## Figures and Tables

**Table 1:** Measured soluble factors in cutaneous wound healing.

Mediator	
**Chemokines**	
MCP-1	Monocyte chemoattractant protein-1
IL-8	Interleukin-8
GRO-α	Growth-related oncogene-α
ENA-78	Epithelial cell-derived neutrophil-activating factor
Eotaxin	Eotaxin
RANTES	Regulated on activation normal T-cell-expressed and secreted
**Cytokines**	
ICAM-1	Intercellular adhesion molecule 1
IL-6	Interleukin-6
IL-1α	Interleukin-1α
GM-CSF	Granulocyte–macrophage colony-stimulating factor
G-CSF	Granulocyte colony-stimulating factor
TNF-α	Tumour necrosis factor-α
VCAM-1	Vascular cell adhesion protein-1
**Growth factors**	
HGF	Hepatocyte growth factor
VEGF-α	Vascular endothelial growth factor-α
FGF-β	Fibroblast growth factor-β
TGF-β	Transforming growth factor-β
PDGF-BB	Platelet-derived growth factor-BB
EGF	Epidermal growth factor

**Table 2:** Culture conditions for keratinocytes supplemented with DFCM.

Conditions	Descriptions
DFCM-KM-1	DFCM-KM acquired by incubating fibroblasts for 1 day
DFCM-KM-2	DFCM-KM acquired by incubating fibroblasts for 2 days
DFCM-KM-3	DFCM-KM acquired by incubating fibroblasts for 3 days
DFCM-FM-1	DFCM-FM acquired by incubating fibroblasts for 1 day
DFCM-FM-2	DFCM-FM acquired by incubating fibroblasts for 2 days
DFCM-FM-3	DFCM-FM acquired by incubating fibroblasts for 3 days
Control	Epilife (keratinocyte-specific medium, KM) alone

**Table 3:** Concentration of calcium chloride in DFCM-KM-3, DFCM-FM-3, and after DFCM-FM-3 dialysis using a dialysis kit.

Conditioned Medium	Calcium Concentration (mmol/L)
DFCM-KM-3	0.06
DFCM-FM-3	1.08
DFCM-FM-3 (dialysis)	0.01

## Data Availability

For Data Transparency, the authors declare that all relevant data are included in the article, and the datasets generated and/or analysed during the study are available within the articles or from the corresponding author on reasonable request. For code availability (Software Application or Custom Code): Data for cell migration and wound area were analysed using NIS Element AR 3.1 software (Nikon).

## Abbreviations

DFCM, dermal fibroblast conditioned medium; ECM, extracellular matrix; KGF, keratinocyte growth factor; GM-CSF, granulocyte–macrophage colony-stimulating factor; IL, interleukin; FGF, fibroblast growth factor; PDGF, platelet-derived growth factor; CM, conditioned medium; ICAM-1, Intercellular adhesion molecule 1; GM-CSF, granulocyte–macrophage colony-stimulating factor; G-CSF, granulocyte colony-stimulating factor; MCP, monocyte chemoattractant protein; GRO, growth-related oncogene; ENA, epithelial cell-derived neutrophil-activating factor; HGF, hepatocyte growth factor; VEGF, vascular endothelial growth factor; bFGF, basic fibroblast growth factor; CK, cytokeratin; P3, Passage 3; BCA, bicinchoninic acid; BSA, bovine serum albumin; SEM, standard error of the mean; ANOVA, analysis of variance.
